# Successful treatment of severe sinusoidal obstruction syndrome despite multiple organ failure with defibrotide after allogeneic stem cell transplantation: a case report

**DOI:** 10.4076/1752-1947-3-6164

**Published:** 2009-03-10

**Authors:** Gerhard Behre, Sebastian Theurich, Maximilian Christopeit, Thomas Weber

**Affiliations:** 1 Department of Internal Medicine IV, Oncology and Haematology, Martin-Luther-University Halle-Wittenberg, Ernst-Grube-Strasse, 06097 Halle, Germany

## Abstract

**Introduction:**

We report a case of sinusoidal obstruction syndrome, a typical and life-threatening complication after allogeneic stem-cell transplantation, successfully treated with defibrotide despite massive multiple organ failure.

**Case presentation:**

A 64-year-old Caucasian woman underwent allogeneic peripheral blood stem-cell transplantation from her human leukocyte antigen-identical sister against aggressive lymphoplasmocytoid immunocytoma. Seven days later, the patient developed severe sinusoidal obstruction syndrome according to the modified Seattle criteria. We initiated treatment with defibrotide. Despite early treatment, multiple organ failure with kidney failure requiring dialysis and ventilator-dependent lung failure aggravated the clinical course. Furthermore, central nervous dysfunction occurred as well as transfusion refractory thrombocytopenia.

**Conclusion:**

As highlighted in our report, defibrotide is the most promising drug in the treatment of the formerly, almost lethal, severe sinusoidal obstruction syndrome to date. This is demonstrated very clearly in our patient. She improved completely, even after renal, cerebral and respiratory failure.

## Introduction

We report a case of sinusoidal obstruction syndrome (SOS), a typical and life-threatening complication after allogeneic stem-cell transplantation, successfully treated with defibrotide despite massive multiple organ failure.

## Case presentation

A 64-year-old Caucasian woman underwent allogeneic peripheral blood stem-cell transplantation from her human leukocyte antigen (HLA)-identical sister against aggressive lymphoplasmocytoid immunocytoma. Conditioning comprised of hyperfractionated total-body irradiation with 4 Gy on day 8 of the transplant, 3 Gy on day 7 (total dose 7 Gy), fludarabine 30 mg/m^2^ on days 7 to 4 (total dose 120 mg/m^2^) and cyclophosphamide 30 mg/kg on days 5 and 4 (total dose 60 mg/kg). Immunosuppression was accomplished by cyclosporine A (CsA) and mycophenolat mofetil. When starting the treatment, our patient had only a few factors associated with increased risk of veno-occlusive disease (VOD): previous treatment with cyclophosphamide, acyclovir prophylaxis during conditioning and female gender. Both, donor and patient were cytomegalovirus immunoglobulin g-positive. The patient had no history of abdominal irradiation or pre-existing liver disease. Thus, we abstained from SOS prophylaxis with heparin or ursodeoxycholic acid following our intern transplant policies.

Three days after transplantation, the patient developed neutropenic fever. Empiric antibiotic therapy with teicoplanin 400 mg daily after a loading dose of 800 mg and piperacillin/combactam 4 g/1 g three times daily were started. The fever persisted for more than 2 days, and voriconazole was started with 200 mg two times daily. Twenty-four hours later, an infection of the permanent central venous catheter was identified as focus. The catheter was removed and clindamycine 600 mg four times daily was added to the antibiotic regimen. Apart from voriconazole, no medication associated with microangiopathia was administered.

**Figure 1:**
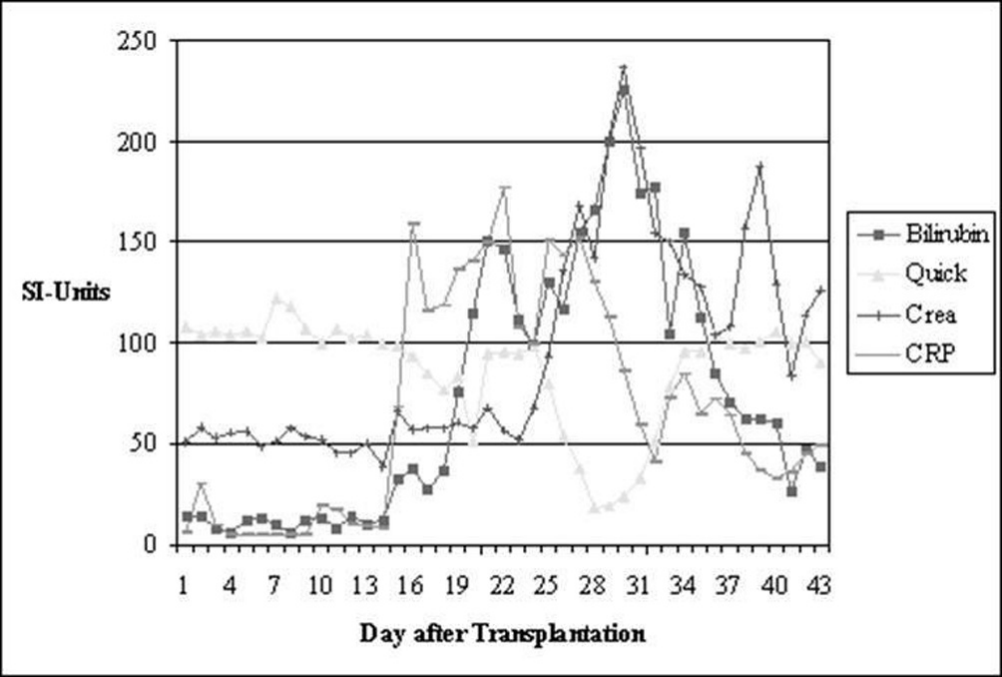
**Course of the paraclinical parameters**. Bilirubin, total serum bilirubin; Quick, Crea, serum creatinine; CRP, C-reactive protein.

On day 7 after transplantation, the patient developed fluid retention. The patient complained of right upper quadrant pain, she gained weight, and her total bilirubin serum levels started to rise (Figure 1). Abdominal ultrasound showed liver enlargement. CsA serum levels were in the normal range. Although ultrasound on day 10 did not show any flow abnormalities of the liver veins, we established the diagnosis SOS according to the modified Seattle criteria [1] with 1) jaundice and bilirubin >34.2 μmol/l; 2) hepatomegaly or right upper quadrant pain; and 3) fluid retention >2% of the initial body mass. We initiated treatment with defibrotide (starting with a dose of 200 mg four times a day, escalated up to 800 mg four times a day on day 13) and 80IU unfractionated heparin/kg/day on day 9. There was no other causal treatment.

**Figure 2:**
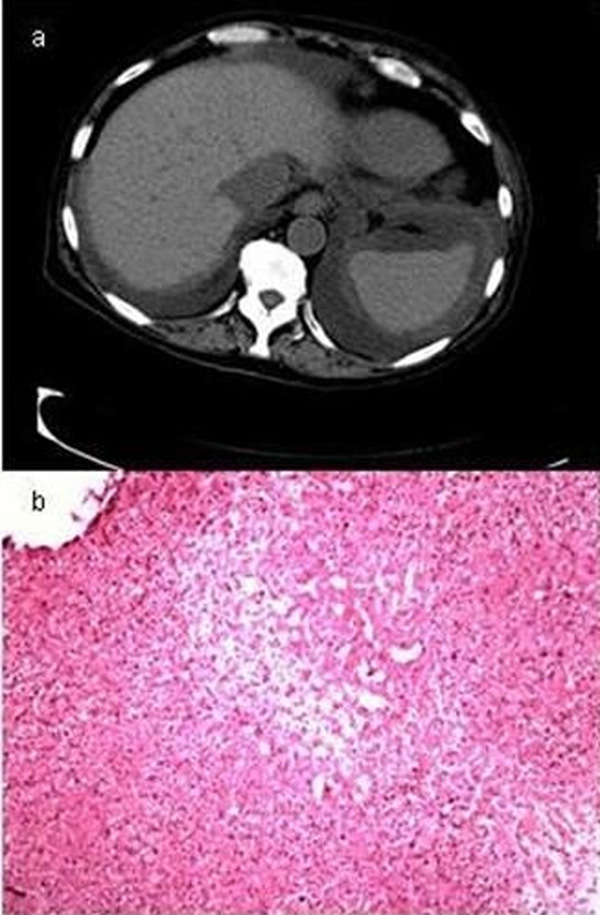
**(a) Computer tomography scan of the abdomen with hepathomegaly and ascites; (b) liver tissue obtained through transjugular liver biopsy, haematoxylin eosin stained with necrosis and inflammatory round-cell infiltration through transjugular liver biopsy**.

Despite early treatment, multiple organ failure (MOF) with kidney failure requiring dialysis and ventilator-dependent lung failure aggravated the clinical course. Symptomatic treatment comprised of dialysis and diuretic therapy with torasemide 10 mg/hour. Lung function was sustained by mechanical ventilation. Furthermore, central nervous dysfunction as well as transfusion refractory thrombocytopenia was observed. We classified this case as severe SOS defined by MOF and according to the criteria of DeLeve [2]. A transjugular liver biopsy performed on day 22 confirmed the diagnosis SOS (Figure 2), with the typical associated histological findings such as sinusoidal congestion with centrolobular necrosis and, later, fibrous obliteration of the hepatic venules and perivenular fibrosis [2,3]. Fifteen days after the onset of the disease, the clinical symptoms vanished and liver enzymes normalized. In a control computer tomography scan of the abdomen, liver size and ascites declined. Defibrotide was ceased on day 59. Because of massive gastrointestinal haemorrhage, we paused defibrotide for 3 days. Finally, complete restitution was achieved.

## Discussion

There is no accepted standard of therapy for severe SOS. Despite thrombolytic therapy with tissue-plasminogen activator (t-PA) or prostaglandin E1 with or without heparin, the mortality of severe VOD has remained about 90%. Most patients die of MOF secondary to SOS. Haemorrhage frequently delimitates treatment [4].

Defibrotide, a single-stranded polydesoxyribonucleotide with specific binding sites on vascular endothelium, was issued to general phase-II single-arm studies. Some of these studies showed encouraging success rates against severe SOS within 35% to 40% [5,6]. This is demonstrated very clearly in our patient. She improved completely, even after renal, cerebral and respiratory failure. It is unlikely that this tremendous improvement was substantially caused by the low-dose heparin infusion.

Defibotide has local antithrombotic and thrombolytic effects in the injured endothelium but lacks systemic anticoagulative effects [7]. Adverse effects are less frequent than with other available treatment options. The anticoagulative effects are probably due to its function as an adenosine receptor antagonist with up-regulation of the endothelium release of t-PA, nitric oxide, prostacyclin, prostaglandin E2 and thrombomodulin, as well as down-regulation of the release of plasminogen activator-inhibitor 1. It also seems to decrease endothelin activity [8]. The reason for the gastrointestinal bleeding in our patient is hard to trace but most likely based on MOF with consecutive coagulopathy.

The reason for the VOD in our case is hard to trace. The initial risk profile of our patient was low. However, persistent fever and concordant treatment with glycopeptides, as in our case, is associated with a higher risk of VOD [1]. Probably the infection and the medication with voriconazole could have acted as a trigger for VOD in our patient.

## Conclusion

As highlighted in our report, defibrotide is the most promising drug in the treatment of the former, almost lethal, severe SOS to date. To increase response to treatment, ongoing investigations focus on the combination of defibritode with other drugs as the inhibitor of glutathione depletion n-acetyl cysteine. In general, management of SOS should be based on prophylaxis: identification of patients at risk, avoidance of SOS-inducing therapies, preventative medical therapy [9] and, finally, therapy of the syndrome.

## Abbreviations

SOS: Sinusoidal obstruction syndrome; HLA: human leukocyte antigen; VOD: veno-occlusive disease; CsA: cyclosporine A; MOF: multiple organ failure.

## Consent

Written informed consent was obtained from the patient for publication of this case report and accompanying images. A copy of the written consent is available for review by the Editor-in-Chief of this journal.

## Competing interests

The authors declare that they have no competing interests.

## Authors' contributions

All authors treated the patient. G.B and TW wrote the manuscript.
